# Recombinant BCG expressing the LTAK63 adjuvant improves a short-term chemotherapy schedule in the control of tuberculosis in mice

**DOI:** 10.3389/fimmu.2022.943558

**Published:** 2022-08-31

**Authors:** Monalisa Martins Trentini, Alex Issamu Kanno, Dunia Rodriguez, Lazaro Moreira Marques-Neto, Silas Fernandes Eto, Ana Marisa Chudzinki-Tavassi, Luciana Cezar de Cerqueira Leite

**Affiliations:** ^1^ Laboratório de Desenvolvimento de Vacinas, Instituto Butantan, São Paulo, Brazil; ^2^ Laboratory Center of Excellence in New Target Discovery (CENTD) Special Laboratory, Instituto Butantan, São Paulo, Brazil; ^3^ Center of Innovation and Development, Laboratory of Development and Innovation, Instituto Butantan, São Paulo, Brazil

**Keywords:** BCG, tuberculosis, recombinant BCG, rBCG-LTAK63, immunotherapy, immuno-therapeutic vaccine

## Abstract

Tuberculosis (TB) is one of the deadliest infectious diseases around the world. Prevention is based on the prophylactic use of BCG vaccine, effective in infants but as protection wanes with time, adults are less protected. Additionally, chemotherapy requires the use of many antibiotics for several months to be effective. Immunotherapeutic approaches can activate the immune system, intending to assist chemotherapy of TB patients, improving its effectiveness, and reducing treatment time. In this work, the recombinant BCG expressing LTAK63 (rBCG-LTAK63) was evaluated for its immunotherapeutic potential against TB. Bacillary load, immune response, and lung inflammation were evaluated in mice infected with *Mycobacterium tuberculosis* (*Mtb*) and treated either with BCG or rBCG-LTAK63 using different routes of administration. Mice infected with *Mtb* and treated intranasally or intravenously with rBCG-LTAK63 showed a reduced bacillary load and lung inflammatory area when compared to the group treated with BCG. In the spleen, rBCG-LTAK63 administered intravenously induced a higher inflammatory response of CD4^+^ T cells. On the other hand, in the lungs there was an increased presence of CD4^+^IL-10^+^ and regulatory T cells. When combined with a short-term chemotherapy regimen, rBCG-LTAK63 administered subcutaneously or intravenously decreases the *Mtb* bacillary load, increases the anti-inflammatory response, and reduces tissue inflammation. These findings highlight the potential of rBCG-LTAK63 in assisting chemotherapy against *Mtb*.

## Introduction

TB is a global infectious disease that affects millions of people each year. While most patients will be clinically asymptomatic and classified as latent TB, it can change to an active form under several conditions. Even with the availability of prophylactic and therapeutic treatments, TB mortality was approximately 1.5 million in 2020 (1). The chemotherapy for TB demands the administration of several antibiotics for at least 6 months, which leads several patients to abandon the treatment after a transitory recovery (1, 2). Incomplete treatment contributes to the emergence of multidrug-resistant TB strains which requires an even more prolonged and complex antibiotic therapy. Although current efforts in prevention and treatment have decreased its prevalence, the situation remains far from being resolved. As a result, new strategies to treat TB patients are constantly under investigation.

The use of vaccines as immunotherapy in the treatment of TB patients is an attractive idea as it can reduce the *Mtb* bacillary load and result in a better clinical outcome. Immunotherapy using vaccines may be especially helpful if combined with chemotherapy (3, 4). While most vaccines in clinical trials were devised as prophylactic, a limited number are also under evaluation in a post-infection immunotherapy regimen. Whole-cell vaccines are among the most advanced ones in clinical trials to which antituberculosis effect are usually associated with an increased Th1 immune response (5–11). rBCG-LTAK63 is a recombinant BCG expressing the LTAK63 adjuvant, the genetically detoxified subunit A of the LT toxin from *Escherichia coli* (12). Immunization of mice with rBCG-LTAK63 induces improved protection against *Mtb* challenge. Protection was associated with an increased production of Th1-related cytokines in immunized mice as well as an increase in innate and long-term immune responses (13). Interestingly, after the *Mtb* challenge, mice immunized with rBCG-LTAK63 showed an increased production of regulatory-related cytokines (TGF-beta and IL-10) in the lungs, resulting in considerable reduction of the inflammation associated with infection (12). We hypothesize that the therapeutic use of rBCG-LTAK63 could induce an improved control over the bacillary load and inflammation caused by *Mtb* infection.

In this context, we have evaluated the effect of rBCG-LTAK63 in the treatment of Mtb-infected mouse models and provide compelling evidence that it can be effective as immunotherapy for TB. Our findings also demonstrate that in a scenario where the antibiotic therapy is shorter – mimicking a discontinued chemotherapy – the immunotherapy with rBCG-LTAK63 can activate the immune system, reduce the Mtb bacillary load and the inflammation associated with the disease.

## Material and methods

### Animals

Six-week-old BALB/c female mice from the Biotério Central of Instituto Butantan were housed and bred in micro isolators adapted to HEPA filtered racks with 12 h light/dark cycles, temperature ranging from 20 to 22°C, and 60% humidity at the animal housing facilities of the Laboratório de Desenvolvimento de Vacinas, Instituto Butantan. This study and protocols were approved by the Ethical Committee on Animal Use of Instituto Butantan (Protocol number: 5135010819).

### *Mtb* intranasal infection

Aliquots of *Mtb* (H37Rv) maintained at –80° C were thawed, and the concentration adjusted to 1.25 x 10^4^ CFU/mL. Groups of 5 mice under mild anesthesia were instilled with 40 µL (500 CFU) into the nostrils with aid of a micropipette. CFU recovered from the lungs of an extra control group (with 5 animals) thirty days after infection was evaluated prior to the beginning of treatment. At this point, the CFU was 1.59 ± 0.52 x 10^6^ CFU/lung (SD).

### Tuberculosis immunotherapy protocols

Frozen aliquots of rBCG-LTAK63 were diluted in PBS 0.05% Tween 80 and 10^6^ CFU/mouse administered through different routes. The native BCG Moreau vaccine (10^6^ CFU/mouse) was administered as control. The immunotherapeutic protocol of infected animals was performed as previously described (14, 15). Briefly, after four weeks of infection, the groups of BALB/c mice were treated either with one or two doses (30 days interval) of BCG or rBCG-LTAK63 administered *via* subcutaneous (SC, 100 µl), intranasal (IN, 40 µl), or intravenous (IV, 100 µl) routes. As control, chemotherapy with antibiotics consisting of rifampicin (RIF, 20 mg/kg) and isoniazid (INH, 50 mg/kg) (Sigma-Aldrich^®^, Merck KGa, St Louis, MO, USA) was given daily by gavage for four weeks. Mice were euthanized four and eight weeks after the immunotherapy, the spleen and/or lung (cranial and median lobes) collected, homogenized, diluted and plated on Middlebrook 7H10 agar (BD Difco™, Detroit, MI, USA), supplemented with 0.5% glycerol (Sigma-Aldrich^®^), 10% OADC (oleic acid-albumin-dextrose-catalase; BBL, Cockeysville, MD, USA), 5 µg/ml of TCH (2-thiophenecarboxylic acid hydrazide, Sigma-Aldrich^®^, a BCG growth inhibitor) and incubated at 37°C and 5% CO_2_ for 2-3 weeks to verify the bacterial load as determined by the number of CFU recovered. To evaluate the combination of chemotherapy plus immunotherapy, 4 weeks after infection, RIF (20 mg/kg) and INH (50 mg/kg) were administered by daily gavage for only 2 weeks (short-term chemotherapy). After 2 weeks interval, one dose of rBCG-LTAK63 (10^6^ CFU/mouse) was administered *via* SC, IN or IV routes. The immune response and bacterial burden were evaluated 8 weeks after the immunotherapy.

### Inflammatory immune response

Cell preparations were done as previously described (16). Briefly, mice were euthanized, and the lungs and spleens collected in ice-cold RPMI-1640 (Sigma-Aldrich^®^). The lungs were digested with DNAse IV (30 μg/mL; Sigma-Aldrich^®^) and collagenase III (0.7 mg/mL; Sigma-Aldrich^®^) for 30-40 min at 37°C. The digested tissue was prepared as single-cell suspensions using 70 μm cell strainers (BD Pharmingen™, San Diego, CA), erythrocytes were lysed with an RBC lysis solution (150 mM NH_4_Cl, 10 mM KHCO_3_ pH 7.4), the cells were washed once and resuspended in RPMI-1640. Cells were counted in a Neubauer chamber using Trypan Blue and adjusted to 1 × 10^6^ cells/mL and distributed in 96-well plates (Corning^®^, NY, USA). The cells were stimulated with anti-CD3 (1 µg/mL, clone: OKT3, BD Pharmingen™) plus anti-CD28 (1 µg/mL, clone: CD82.2, BD Pharmingen™) for 48 h at 37 °C and 5% CO_2_. The supernatant was collected, and the concentration of cytokines determined by Cytometric Bead Array (BD Pharmingen™) using the Mouse Th1/Th2/Th17 Cytokine Kit.

Additionally, in order to determine the phenotype of T cells in the spleen and lungs, the samples were stained with: anti-CD4-PercP antibody (clone: RM4-5, BD Pharmingen™) and anti-CD3-APC.Cy7 antibody (clone: 17A2, BD Pharmingen™) for 30 min. Then, the cells were washed, fixed, and permeabilized using the Mouse Cytofix/Cytoperm Kit (BD). The cells were further incubated for 30 min with anti-TNF-α-PE (clone: MP6-XT2, BD Pharmingen™), anti-IFN-γ-APC (clone: XMG1.2, BD Pharmingen™), anti-IL-17-BV421 (clone: TC11-18H10, BD Pharmingen™), and anti-IL-10-PE.Cy7 (clone: JESS-16E3, BD Pharmingen™).

Spleen and lung cells were also evaluated for their T regulatory phenotype by staining with Mouse Th17/Treg Phenotyping Kit (BD Pharmingen™). The cells were incubated with anti-CD4-PercP antibody (clone: RM4-5) andanti-CD25-PE antibody (clone: PC619) (BD Pharmingen™) for 30 min. Then, the cells were washed, fixed, and permeabilized using the Mouse Foxp3 Cytofix/Cytoperm Kit (BD). The cells were further incubated for 50 min withanti-FoxP3-AlexaFluor647 antibody (clone: MF23, BD Pharmingen™). Data were acquired on a FACSCanto II flow cytometer (BD Pharmingen™) and analyzed using the FlowJo 8.7 software.

### Immunopathological evaluation of the lungs

After infection and treatments, the right caudal lung lobes of mice were collected and fixed in 10% paraformaldehyde (Labsynth^®^, Diadema, SP). Fixed tissues (5 µm thick) were placed onto glass slides and stained with hematoxylin and eosin (H&E). The intensity of lung inflammation was evaluated according to (17). In brief, H&E-stained sections were photographed at 40 x magnification using a microscope (Axio Imager M2 Zeiss) coupled to a digital camera (Axiocam MRm Zeiss). Image analysis software (ImageJ, National Institutes of Health, USA) was used to determine the pulmonary area affected. Briefly, five images at 40 x magnification per lung lobule, totaling 25 images per group, were randomly selected, and analyzed for the qualitative evaluation of the cell infiltrate and intra-alveolar regions (17–19). To measure the areas of interest, the images were transformed into 8-bit and treated with threshold and percentage of the measured area. For leukocyte counting, the Color Deconvolution 2 plugin was used to visualize and separate nuclei from the cytoplasm. For cell counting, the Cell Counter plugin was used. This analysis is used to facilitate the differential counting of segmented and mononuclear nuclei.

### Statistical analysis

The data were imported to Excel (version 14.3.4, 2011) and analyzed using Graphpad Prism Software 6.0. Statistical analysis was performed by one-way ANOVA followed by Bonferroni test; p values < 0.05 was considered statistically significant. Values are reported as the mean ± SD.

## Results

### rBCG-LTAK63 immunotherapy reduces *Mtb* bacillary load

**Figure 1:**
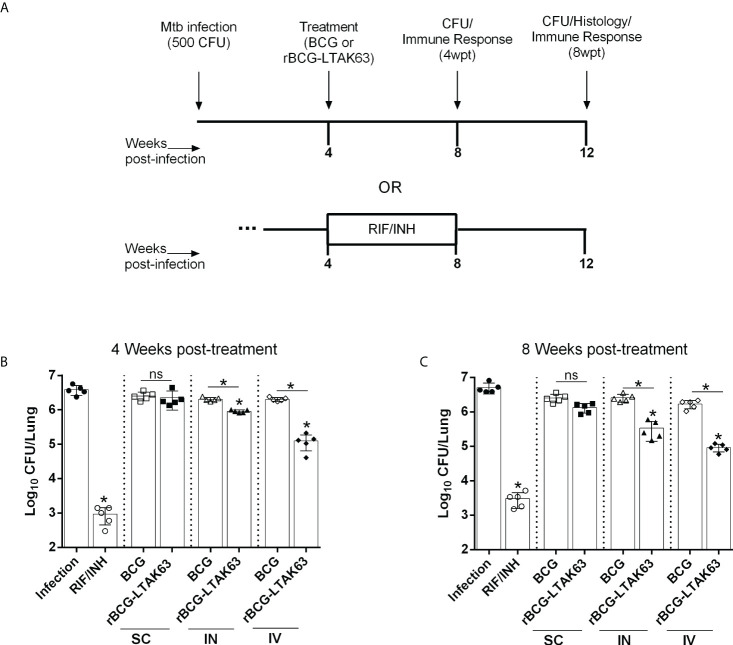
Therapeutic effect of rBCG-LTAK63 vaccine in Mtb-infected mice. **(A)** BALB/c mice were infected with Mtb (500 CFU/IN) and treated 4 weeks later with a single of BCG or rBCG-LTAK63 vaccine delivered by the subcutaneous (SC), intranasal (IN) or intravenous (IV) route. As a control, one group received rifampicin and isoniazid daily given by gavage for 4 weeks (RIF/INH). Four weeks **(B)** or eight weeks **(C)** post-treatment (4wpt or 8wpt), the lungs of these animals were collected and homogenized. Serial dilutions were plated onto 7H10 agar plates containing TCH (a BCG growth inhibitor) to assess Mtb CFU. Statistical differences were determined by one-way ANOVA with a Bonferroni test. *p values ≤ 0.05 were considered statistically significant and ns, not significant. Asterisks over the columns refer to the comparison with the infection group. Results are represented by the means ± SD of the CFU recovered in the cranial and median lung lobes from the groups of mice (n=5/group).

To determine the immunotherapeutic potential of rBCG-LTAK63, BALB/c mice were infected with *Mtb* and treated with a single dose of BCG or rBCG-LTAK63 administered *via* the SC, IN, or IV routes after 4 weeks of infection. The infection of Mtb was performed using the intranasal route with 500 CFU/mouse to mimic the natural infection route, as previously described (20) (**Supplementary Figure 1**). Four weeks after treatment with rBCG-LTAK63, either by the IN or IV routes, showed significantly reduced bacillary load (**Figures 1A, C**). Furthermore, the treatments also induced reduced bacterial load in the spleens (**Supplementary Figure 2**). The complete chemotherapy with INH (50 mg/kg) and RIF (20 mg/kg) administered daily by gavage for 4 weeks was used as positive control. Neither BCG treatment by any route nor rBCG-LTAK63 administered SC were able to reduce the bacillary load (**Figure 1B**). Additionally, we investigated whether this reduction would persist for longer periods after the treatment. At eight weeks after treatment the immunotherapy with rBCG-LTAK63 *via* IN or IV also showed a reduced *Mtb* bacillary load (**Figures 1C, D**). Again, neither rBCG-LTAK63 administered SC nor BCG by any route showed reduction in bacillary load. Comparison of rBCG-LTAK63 administered by different routes demonstrate that the IV route resulted in a greater reduction in the bacillary load than the IN route (**Figures 1B, D**). On the other hand, two doses of rBCG-LTAK63 did not show therapeutic effectiveness in any administration route (IV, IN, or SC) (**Supplementary Figure 3**).

Notably, the group treated with antibiotics exhibited reduced effectiveness over time, which is not observed in the rBCG-LTAK63 vaccine-treated group; that is, in the lungs of animals treated with antibiotics, there is a small increase in bacillary load with time that is not observed in the group treated with rBCG-LTAK63 (**Supplementary Figure 4**).

**Figure 2:**
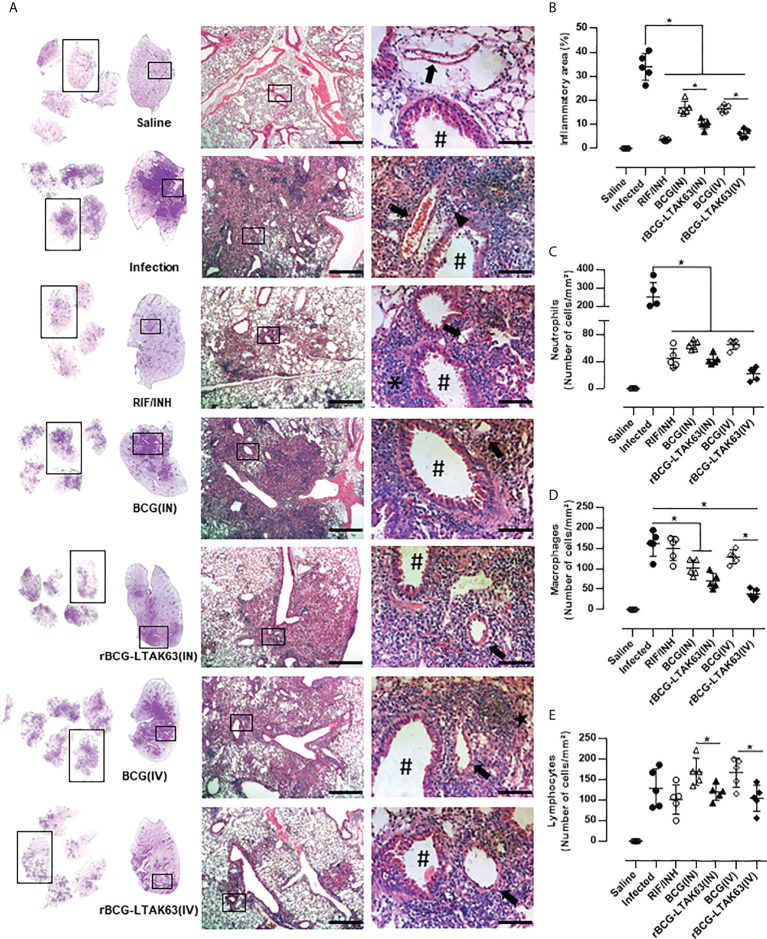
Immunotherapy with rBCG-LTAK63 vaccine decreases cellular infiltration in the lungs of *Mtb*-infected mice. **(A)** Representative histopathology of lungs from naïve mice (Saline), infected with *Mtb* and not treated (Infection), treated with antibiotics for 4 weeks (RIF/INH) or treated with BCG or rBCG-LTAK63 delivered intranasally (IN) or intravenously (IV), at 1x, 5x and 20x magnification. Representative images at 40x magnification show the difference in inflammatory process between treatments, neutrophilic infiltrate (arrowhead), interalveolar macrophage (star) and lymphocyte (asterisk), arteriole (arrow) and bronchioles (hashtag). **(B)** Lung inflammation scores are presented as the mean percentage of inflammation area (mm²) for each mouse (n=5/group). The cellular infiltrate in the lung sections was classified in neutrophils **(C)**, macrophages **(D)** and lymphocytes **(E)** and presented as cell counts per mm². Lung sections were stained with H&E (bar, 200 µm (5x) and/20 µm (20x). Statistical differences were determined by one-way ANOVA with a Bonferroni test. *p < 0.05.

To evaluate the degree of inflammation, the lungs of infected mice treated with BCG or rBCG-LTAK63 were processed for histopathological examination. Mice infected with *Mtb* (Infection group) developed widespread and diffuse pneumonia, as well as an inflammatory infiltrate surrounding the blood vessels and airways (**Figure 2A**). In comparison, mice treated with the BCG vaccine (IN and IV routes) developed pneumonia patches and had more severe perivascular and peribronchiolar inflammatory infiltrates than animals treated with rBCG-LTAK63 or conventional antibiotics (RIF/INH) (**Figure 2A**). Surprisingly, mice treated rBCG-LTAK63 (IN and IV routes) showed decreased lung inflammation (**Figure 2B**) with reduced neutrophils, macrophages and lymphocytes efflux when compared with the infected animals or mice that received BCG (**Figures 2C–E**).

### rBCG-LTAK63 therapy modulates the inflammatory response induced by *Mtb*

**Figure 3:**
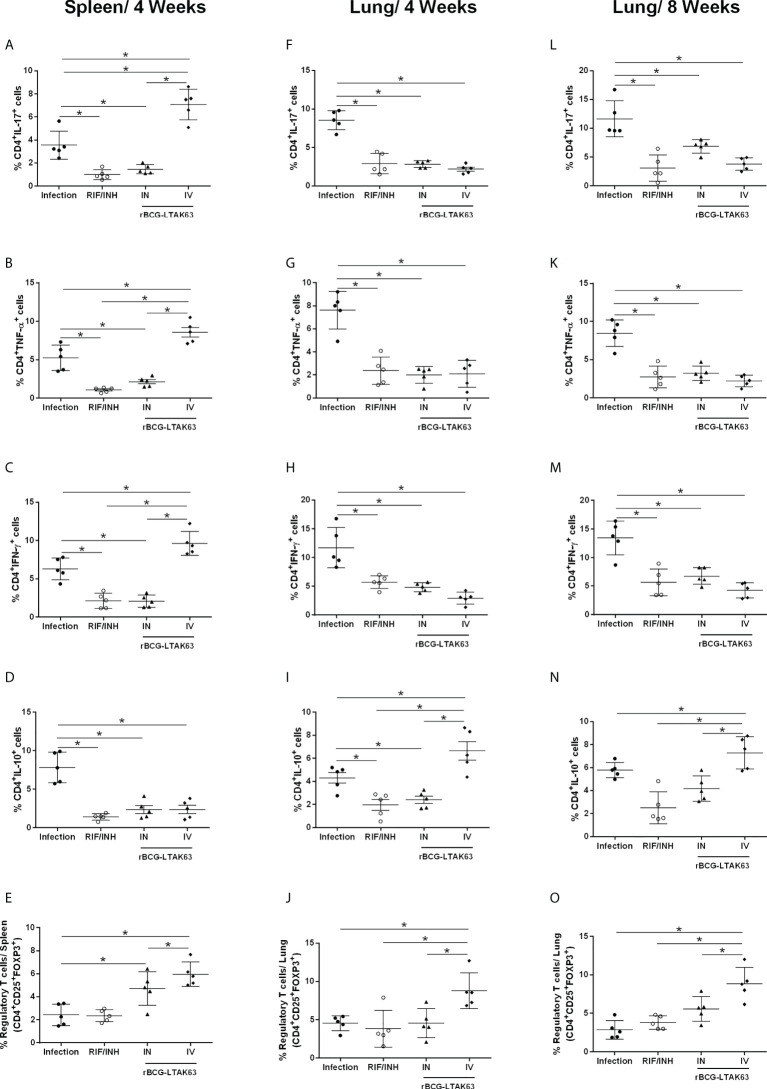
Immunotherapy with rBCG-LTAK63 modulates proinflammatory and regulatory immune responses in *Mtb*-infected mice. BALB/c mice were infected with *Mtb* (Infection) and treated with RIF/INH (*via* gavage for 4 weeks) or a single dose of rBCG-LTAK63 delivered intranasally (IN) or intravenously (IV). The immune response in the spleen and lungs was evaluated four and eight weeks post-treatment (4wpt and 8wpt). The number of CD4^+^ T cells producing IL-17, **(A, F, L)** TNF-α **(B, G, K)**, IFN-γ **(C, H, M)** and IL-10 **(D, I, N)** in these organs was determined by staining with mouse monoclonal antibodies anti-CD3-APC.CY7, anti-CD4-PercP and anti-IL17-BV421, anti-TNF-PE, anti-IFN-APC or anti-IL-10-PE.Cy7. The percentage the regulatory T cells was determined by staining with mouse monoclonal antibodies anti-CD4-PercP and anti-CD25-PE and anti-FoxP3-APC **(E, J, O)**, and analyzed using Flow Cytometer FACS Canto II and FlowJo 8.7 software. Statistical differences were determined by one-way ANOVA with a Bonferroni test. *p values ≤ 0.05 were considered statistically significant. Asterisks over the columns refer to the comparison with the infection group. Results are represented by the means ± SD of the percentage of CD4+ T cells (for IL-17, TNF-α, IFN-γ and IL-10) or the percentage of regulatory T cells in the CD4^+^ population (n=5/group).

Since the immunotherapy with rBCG-LTAK63 decreased bacillary load and the cellular infiltrate in the lungs of *Mtb*-infected mice, we evaluated the profile of inflammatory and regulatory CD4^+^ T cells in the lungs and spleens following the IN or IV treatment with rBCG-LTAK63 (the gating strategy is shown in **Supplementary Figure 5**). Four and eight weeks after treatment, the mice that received rBCG-LTAK63 IV showed an increased presence of splenic CD4^+^ IL-17^+^ (**Figure 3A**) and CD4^+^ TNF-α^+^ (**Figure 3B**). The increase of CD4^+^IFN-γ^+^ in the spleen was not statistically significant (**Figure 3C**). At the same time, a reduced presence of these cells was observed in the lungs. This was observed at four (**Figures 3F–H**) and eight weeks after treatment (**Figures 3L, M**), as determined by IL-17^+^ (**Figures 3F, L**), TNF-α^+^ (**Figures 3G, K**) and IFN-γ^+^ (**Figures 3H, M**) CD4^+^ T cells. Interestingly, the lower inflammatory response in the lungs from the rBCG-LTAK63 IV treatment was accompanied by an increased number of regulatory T cells (CD4^+^CD25^+^FoxP3^+^) in the spleen and lungs (**Figures 3E, J, O**). Furthermore, increased CD4^+^IL-10^+^ was observed in the lungs in comparison to the Infection group (**Figures 3D, I, N**). These findings show that the IV treatment with rBCG-LTAK63 increases the inflammatory response in the spleen and decreases inflammation in the lungs, which may be correlated with the increase in regulatory T cell response.

When two doses of rBCG-LTAK63 were administered the main effect observed was in IL-17 production through the IN and IV routes. Additionally, the IN route showed a significant increase in IFN-γ produced by lung cells (**Supplementary Figure 6**). The lung mucosal route will induce mostly IL-17, due to the Lung associated Lymphoid Tissue which produces more IL-6 and TGF-b.

### Association of chemotherapy and immunotherapy with rBCG-LTAK63

The antibiotic therapy for TB can last several months and is one of the most common reasons for its discontinuation. After showing that rBCG-LTAK63 may be effective in reducing bacillary load and lung inflammation associated with the infection, we tested whether its combination with a reduced regimen of antibiotic therapy could improve the resolution of the infection (e.g., lower bacillary loads and/or disease-related inflammation).

**Figure 4:**
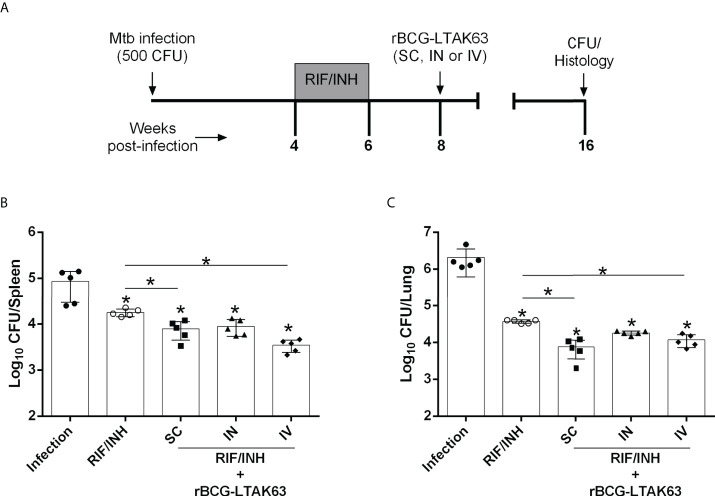
Combination of chemotherapy and rBCG-LTAK63 in the treatment of Mtb infection. **(A)** Schematical representation of the combination of chemotherapy with rBCG-LTAK63. BALB/c mice were infected with Mtb (500 CFU/IN) and treated 4 weeks later with rifampicin and isoniazid (RIF/INH) for 2 weeks. After two weeks interval rBCG-LTAK63 was given *via* SC, IN or IV routes. Eight weeks after treatment, the spleens and lungs of these animals were collected and homogenized. Serial dilutions were plated onto 7H10 agar plates containing TCH (a BCG growth inhibitor) to assess Mtb CFU. Bacillary load in the spleens **(B)** and lungs **(C)** of Mtb-infected mice treated with combination chemotherapy (RIF/INH) and rBCG-LTAK63. Statistical differences were determined by one-way ANOVA with a Bonferroni test. *p values ≤ 0.05 were considered statistically significant. Asterisks over the columns refer to the comparison with the infection group. Results are represented by the means ± SD of the CFU recovered in the cranial and median lung lobes from the groups of mice (n=5/group).

To test this hypothesis, antibiotics were administered for only 2 weeks (half treatment) and two weeks after the end of chemotherapy, they received a single dose of rBCG-LTAK63 (SC, IN or IV route) (**Figure 4A**). Bacillary load in the spleen (**Figure 4B**) and lungs (**Figure 4C**) was determined 8 weeks later. As expected, the reduced chemotherapy decreased the bacillary load (~1.5 log_10_ reduction), but less efficiently than the full 4 weeks regimen (~ 3 log_10_ reduction). As hypothesized, the combination of antibiotic and immunotherapy with rBCG-LTAK63 (SC and IV routes) exhibited a substantial decrease in bacillary load in the spleen and lungs, as compared to the group that received only antibiotics (**Figures 4B, C**). Surprisingly, chemotherapy plus rBCG-LTAK63 administered *via* the IN route did not show any further reduction in comparison to chemotherapy only.

**Figure 5:**
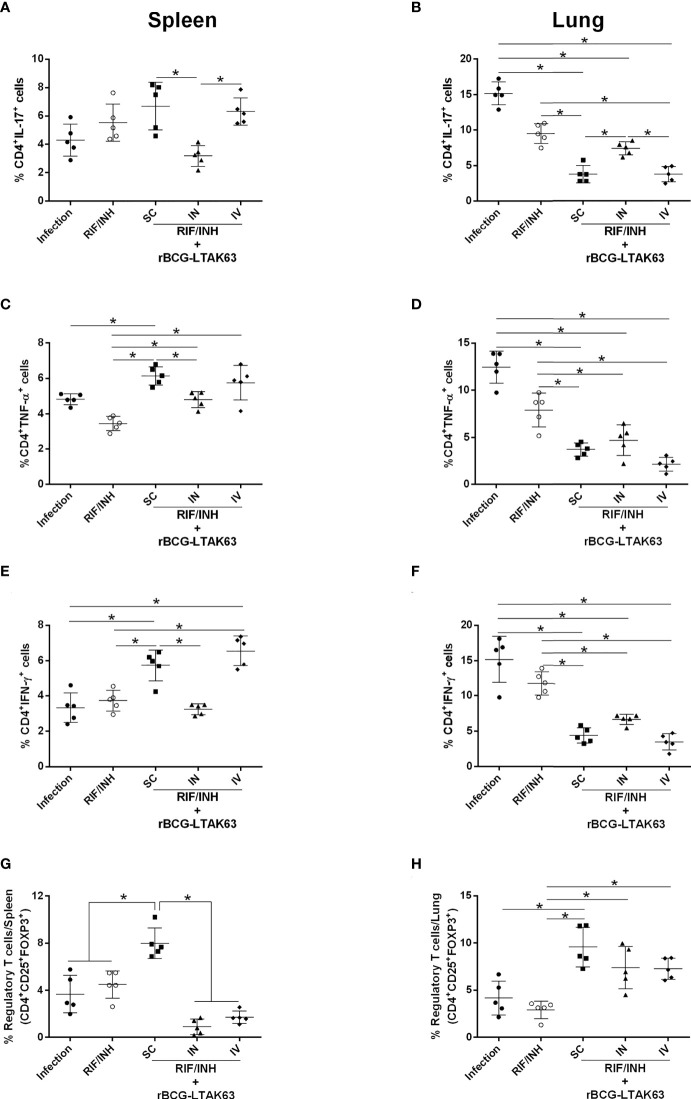
Reduced chemotherapy in combination with rBCG-LTAK63 modulates the immune response in the spleen and lungs of *Mtb*-infected mice. BALB/c mice were infected with *Mtb* (500 CFU/IN) and treated 4 weeks later with rifampicin and isoniazid (RIF/INH) for 2 weeks. After two weeks interval, rBCG-LTAK63 treatment was administered *via* SC, IN or IV routes. Eight weeks after treatment, spleens and lungs were recovered, and the number of CD4+ T cells producing IL-17 **(A, B)**, TNF-α **(C, D)** and IFN-y **(E, F)** in these organs was determined by staining with mouse monoclonal antibodies antiCD3-APC.CY7, anti-CD4-PercP and anti-IL17-BV421, anti-TNF-PE, anti-IFN-APC. Regulatory T cells **(G–H)** were stained using mouse monoclonal antibodies anti-CD4-PercP, anti-CD25-PE and anti-FoxP3-APC, and analyzed using Flow Cytometer FACS Canto II and FlowJo 8.7 software. Statistical differences were determined by one-way ANOVA with a Bonferroni test. *p values ≤ 0.05 were considered statistically significant. Asterisks over the columns refer to the comparison with the infection group. Results are represented by the means ± SD (n=5/group).

**Figure 6:**
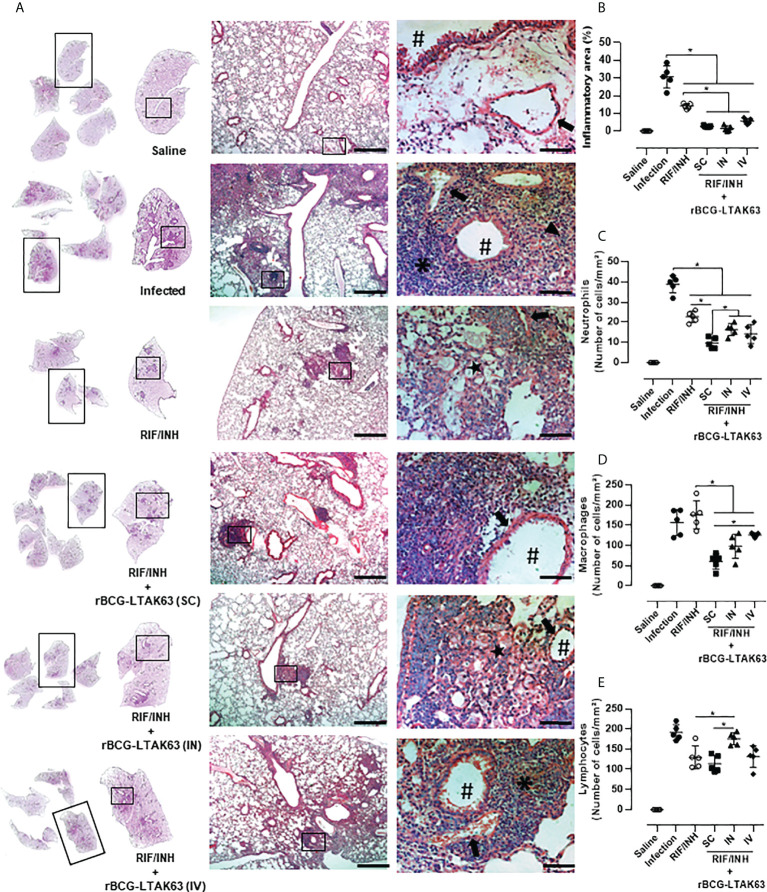
Histopathological analysis of the lungs of mice treated with combination antibiotics (RIF/INH) and rBCG-LTAK63 by different inoculation routes. **(A)** Representative histopathology of lungs from naïve mice (Saline), infected with Mtb and not treated (Infection), treated with antibiotics for 2 weeks (RIF/INH) or treated with combination antibiotics and rBCG-LTAK63 delivered subcutaneously (SC), intranasally (IN) or intravenously (IV) at 1x, 5x and 20x magnification. Representative images at 40x magnification show the difference in inflammatory process between treatments, neutrophilic infiltrate (arrowhead), interalveolar macrophage (star) and lymphocyte (asterisk), arteriole (arrow) and bronchioles (hashtag). **(B)** Lung inflammation scores are presented as the mean percentage of inflammation area (mm²) for each mouse (n=5/group). The cellular infiltrate in the lung sections was classified in neutrophils **(C)**, macrophages **(D)** and lymphocytes **(E)** and presented as cell counts per mm². Lung sections were stained with H&E (bar, 200 µm (5x) and/20 µm (20x). Statistical differences were determined by one-way ANOVA with a Bonferroni test. *p < 0.05.

The immune response assessed by the production of inflammatory cytokines by spleen cells from these groups revealed higher levels of TNF-α when rBCG-LTAK63 was delivered by any route, higher IFN-γ when SC and IV was used and higher IL-17 through SC delivery (**Figures 5A, C, E**). In lung cells, a decrease in the production of these cytokines was observed especially when rBCG-LTAK63 was administered *via* SC and IV (**Figures 5B, D, F**). Interestingly, one combination (antibiotics and rBCG-LTAK63 SC route) induced an increase in the number of regulatory T cells in both the spleen and lungs (**Figures 5G, H**). The intravenously treated group also exhibited an increase in the number of regulatory T cells in the lungs. Accordingly, the lungs of these animals (chemotherapy plus rBCG-LTAK63 SC or IN route) showed reduced pneumonia and had fewer perivascular and peribronchiolar inflammatory infiltrates when compared to the Infection group or to the group treated with antibiotics (half regimen) (**Figure 6A**). Moreover, the cellular infiltrate was less prominent as demonstrated by the reduced lung inflammatory area (**Figure 6B**). The shorter chemotherapy regimen plus rBCG-LTAK63 (all 3 routes) induced lower inflammatory area also in comparison to the antibiotic group. Moreover, different routes of administration appear to recruit distinct numbers of neutrophils, macrophages, and lymphocytes. rBCG-LTAK63 administered IV resulted in higher number of neutrophils and macrophages in comparison to SC (**Figures 6C, D**). On the other hand, the number of lymphocytes was higher in the IN group in comparison to SC (**Figure 6E**). These results suggest that the immunotherapy with rBCG-LTAK63 can complement a reduced regimen of chemotherapy resulting in lower bacillary loads and lung inflammation.

## Discussion

In this study, we showed the immunotherapeutic potential of rBCG-LTAK63 in a *Mtb*-infected murine model. rBCG-LTAK63 induces an inflammatory response in the spleens combined with a regulatory response in the lungs, and results in reduced bacillary loads. A second dose of rBCG-LTAK63 increased the immune response but did not reduce bacterial burden. This result is expected since most live vaccines are administered in only one dose due to persistence in the organism. BCG is not effective, probably due to the previous presence of *Mtb* before treatment. The differential effect seen with rBCG-LTAK63 could be due to the adjuvant effects of the toxin derivative.

The immunotherapy with rBCG-LTAK63 can be further combined with conventional antibiotics aiming at reducing the time of chemotherapy required. Other immunotherapeutic strategies commonly used include vaccines in association with chemotherapy (5–7, 21–23). Importantly, these candidates demonstrate increased Th1 responses also as prophylactic vaccines (24–26). The *M. vaccae*, RUTI, MIP and VPM1002 are candidates in clinical trials as prophylactic and therapeutic vaccines. They can activate the Th1-related immune response and work in synergy with antibiotics to reduce the bacterial burden. A common feature in these vaccines is the requirement of two or more doses to control the *Mtb* infection.

In our studies, we did not combine immunotherapy and chemotherapy at the same time. Since rBCG-LTAK63 is a live vaccine, this feature is important for the protection obtained and concomitant use of antibiotics could compromise its efficacy. Striking differences of live and killed BCG have been reported (27, 28). Here, after a shorter use of conventional antibiotics, a single dose of rBCG-LTAK63 was administered. Through this approach, an additional reduction in *Mtb* bacillary load was observed suggesting that this scheme may compensate treatment abandonment; the long time required to cure a patient is regarded as one of the major obstacles to the success of TB treatment (29, 30). This may be especially important since the immunotherapy alone with rBCG-LTAK63 prevented the expansion of *Mtb* bacillary load. When only the antibiotic was used, there was an increase in CFU after cessation of treatment. On the other hand, rBCG-LTAK63 maintained the CFU stable for at least 8 weeks after treatment. This is probably related to its activity on the immune system cells that will continue to act even if the treatment is interrupted, while the antibiotic needs a minimum concentration (bioavailability) to maintain its therapeutic effect. Further investigation will determine whether the vaccine can impair the replication of *Mtb* for a longer time.

The immunotherapy with rBCG-LTAK63 resulted in a considerable increase of pro-inflammatory cytokines, such as TNF-α, IL-17 and IFN-γ, in the spleens. On the other hand, there was a decrease of these in the lungs. Immunotherapy of TB is based on the induction of immune activity and suppression of immune responses, e.g., excessive inflammation, which otherwise may cause tissue damage (31). When vitamin D (1α, 25-dihydroxy-vitamin D3) is administered in the immunotherapy of TB, there is a regulation of the pro-inflammatory response associated with bacterial clearance and decrease of pathological lesions in the lungs (32, 33). Anti-inflammatory cytokines, such as IL-10 and TGF-β counteract pro-inflammatory-mediated effects. Interestingly, we observed an increased number of the regulatory T cells and CD4^+^IL-10^+^ in the lungs of mice treated IV with rBCG-LTAK63. Coincidently, as a prophylactic vaccine, the immunization with rBCG-LTAK63 also induces an increase in IL-10 and TGF-β in the lungs of mice after *Mtb* challenge (12). Furthermore, a balance of pro and anti-inflammatory responses is associated with the differentiation of T helper subsets during the *Mtb* infection and with disease outcome (34, 35).

Our findings demonstrate that immunotherapy with rBCG-LTAK63 reduces both bacterial burden and pathology in the lungs when administered *via* parenteral (IV) or respiratory routes (IN), while the combination with chemotherapy was effective when administered SC or IV. Several studies reported that parenteral immunotherapies or immunizations fail to control TB, a fact related to poorly induced Ag-specific T cells in the lungs (36, 37). This indicates that Ag-specific immune responses may be induced by either route of administration with rBCG-LTAK63. Accordingly, previous studies have shown a high number of Ag-specific T cells in the spleen and lungs of rBCG-LTAK63-immunized mice (12, 13).

In our results, the sole use of rBCG-LTAK63 through the IN and IV routes (without chemotherapy) was more efficient in reducing the TB burden than SC. It’s known that immune responses are compartmentalized and the IN administrations may benefit from direct stimulation of local immune response, while IV would benefit from its faster (IV facilitating delivery to several organs including the lungs) and stronger stimulation (inducing systemic and local immune responses). On the other hand, although the SC route will induce systemic responses, the priming process is slower when compared to the IV route (38). When we combined immunotherapy and chemotherapy, IV and SC, but not IN, reduced even further the bacterial load. Our data showed that the immune response (IL-17, TNF-α, and IFN-γ) in the lungs directly correlates with the Mtb load, while in the spleens it correlates better with the immunotherapy. This divergence may be due to the chemotherapy effect, which boosts systemic T cell responses (as dead bacilli may be processed and properly presented to lymphocytes) and may explain why systemic immunotherapy works better than the local approach.

Severe TB pathology is associated with the uncontrolled secretion of pro-inflammatory cytokines and chemokines, extensive neutrophilic infiltration, and intensified T cell responses, especially Th1 responses (39, 40). In this study, we demonstrated that immunotherapy with rBCG-LTAK63 results in neutrophil efflux control with smaller inflammatory foci mainly constituted by mononuclear/lymphocytic cells. This may be important in reducing tissue damage, enabling enough influx to generate a lymphocytic-enriched granuloma and could reduce intracellular *Mtb* niches in the lungs (41, 42). Here we show that rBCG-LTAK63 immunotherapy induced a timely modulation of the pro and anti-inflammatory responses and was effective in reducing *Mtb* bacillary loads. Although histology highlights the effect of treatment in the control of inflammation/pathology by rBCG-LTAK63, it is not possible to identify cell subsets. Future studies (with more specific techniques such as immunohistochemistry of flow cytometry) can further clarify the specific mechanism by which rBCG-LTAK63 decreases the progression of TB pathogenesis, and whether immunotherapy with rBCG-LTAK63 would also be effective in previously immunized recipients or those exposed to environmental mycobacteria. Ultimately, immunotherapy can be combined with conventional antibiotics being especially relevant to tackle non-adherence to conventional chemotherapy.

## Data availability statement

The original contributions presented in the study are included in the article/**Supplementary Material**. Further inquiries can be directed to the corresponding author.

## Ethics statement

The animal study was reviewed and approved by Ethical Committee on Animal Use of Butantan Institute (Protocol number: 5135010819).

## Author contributions

MT, DR, AK and LL conceived and designed the experiments; MT, LM-N and SE performed the experiments and collected data; MT, LM-N, DR, AK, SE, AC-T and LL processed and analyzed the data; MT, LM-N, DR, AK, SE and LL wrote the manuscript, and all authors critically revised the manuscript. All authors contributed to the article and approved the submitted version.

## Funding

We acknowledge the support from FAPESP (Projects 2017/24832-6, 2019/06454-0 and 2019/02305-0) and Fundação Butantan.

## Conflict of interest

LL has a patent application on the use of rBCG-LTAK63 as vaccine against Mtb.

The remaining authors declare that the research was conducted in the absence of any commercial or financial relationships that could be construed as a potential conflict of interest.

## Publisher’s note

All claims expressed in this article are solely those of the authors and do not necessarily represent those of their affiliated organizations, or those of the publisher, the editors and the reviewers. Any product that may be evaluated in this article, or claim that may be made by its manufacturer, is not guaranteed or endorsed by the publisher.
