# Supplementary material for: Recombinant BCG expressing the LTAK63 adjuvant improves a short-term chemotherapy schedule in the control of tuberculosis in mice
Source: Front Immunol. 2022 Aug 31;13:943558. doi: 10.3389/fimmu.2022.943558 (PMC9471321; doi:10.3389/fimmu.2022.943558)
Supplement: Supplementary file 1 [file DataSheet_1.docx]

Supplementary Material


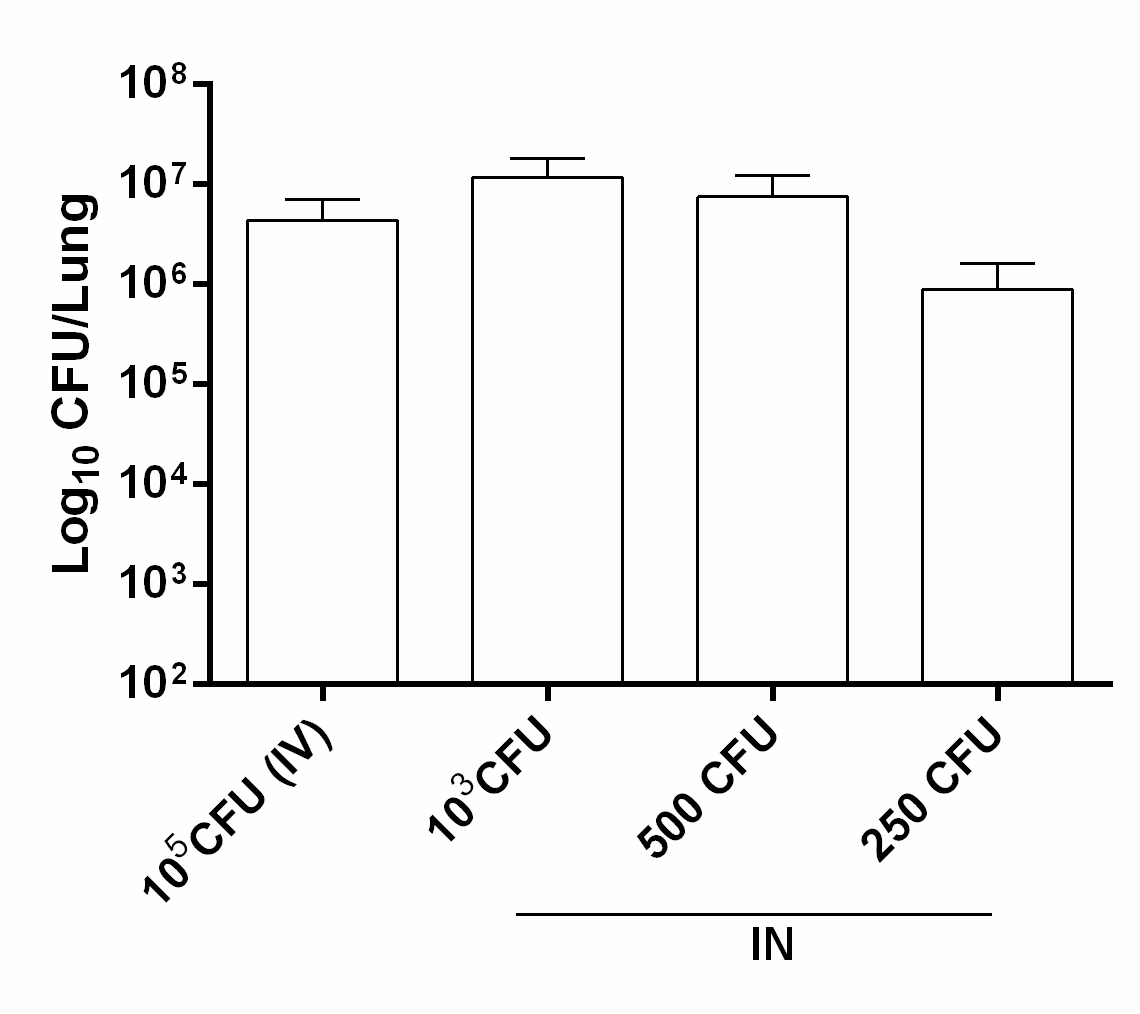


**Supplementary Figure 1.** Standardization of the dose of *M. tuberculosis* infection by the intranasal route. BALB/c mice were infected with *Mtb* by intravenous (10^5^ CFU/ 100 µl) or intranasal (10^3^, 500 and 250 CFU/ 40 µl) route. After 30 days of infection, the bacillary load of the lungs was quantified to comparison between the routes of infection. Results are represented by the means ± SD (n=5/group).

**Supplementary Figure 2.** Therapeutic effect of rBCG-LTAK63 vaccine in the spleens of Mtb-infected mice. BALB/c mice were infected with Mtb (500 CFU/IN) and treated 4 weeks later with a single of rBCG-LTAK63 vaccine delivered by the intranasal (IN) or intravenous (IV) route. As a control, one group received rifampicin and isoniazid daily given by gavage for 4 weeks (RIF/INH). Four weeks post-treatment, the spleens of these animals were collected and homogenized. Serial dilutions were plated onto 7H10 agar plates containing TCH (a BCG growth inhibitor) to assess Mtb CFU. Statistical differences were determined by one-way ANOVA with a Bonferroni test. * *p* values ≤ 0.05 were considered statistically significant. Asterisks over the columns refer to the comparison with the infection group. Results are represented by the means ± SD of the CFU recovered in the spleens from the groups of mice (n=5/group).



**Supplementary Figure 3.** Therapeutic potential with two doses of the rBCG-LTAK63 vaccine against *Mtb* infection. (A) BALB/c mice were infected with *Mtb* (500 CFU/IN); after 4 weeks the animals were treated with two doses of rBCG-LTAK63 by the SC, IN or IV routes, with four weeks intervals. Eight weeks after the second doses, the lungs of *Mtb* infected were collected. (B) Bacillary load in the lungs of *Mtb* infected mice treated or not with two doses of rBCG-LTAK63. Statistical differences were determined by one-way ANOVA with a Bonferroni test. *p* values ≤ 0.05 were considered statistically significant. ns = not significant. Results are represented by the means ± SD (n=5/group).





**Supplementary Figure 4.** Bacterial burden variation between 4 and 8 weeks after treatment. BALB/c mice were infected with *Mtb* (500 CFU/IN) and treated 4 weeks later with a single dose of rBCG-LTAK63 delivered by subcutaneous (SC), intranasal (IN) or intravenous (IV) routes. As a control, one group received rifampicin and isoniazid daily by gavage for 4 weeks (RIF/INH). CFU recovered at 4 weeks was compared to that obtained at 8 weeks and the fold changes are shown. Results are represented by the means ± SD of the fold-change in CFU recovery between the groups of mice (n=5/group).


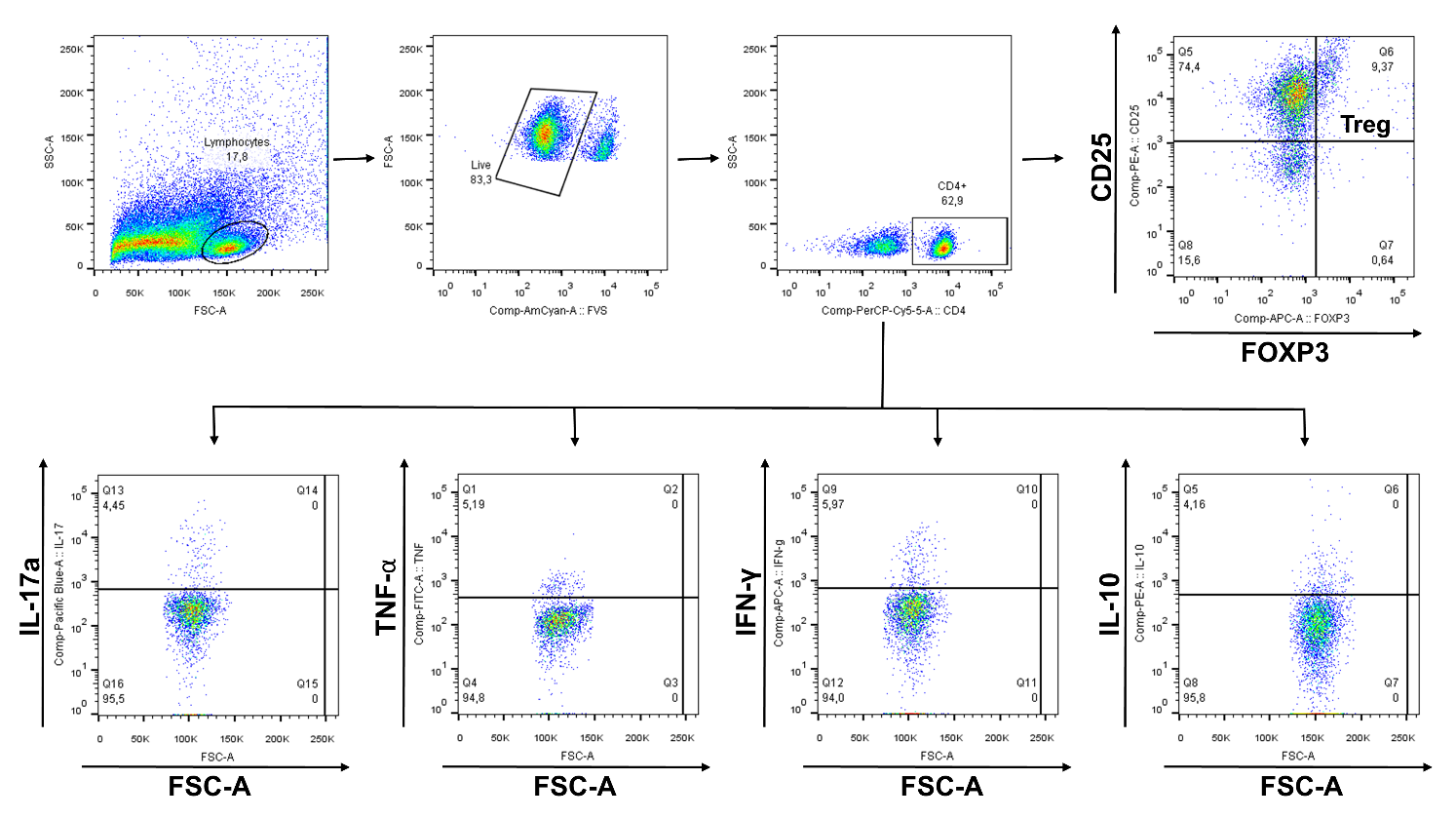


**Supplementary Figure 5.** Gating strategy to identify lymphocytes and their cytokines. Spleen and lung cell suspensions were gated for lymphocytes by FSC (Forward scatter) and SSC (Side scatter) and then live/dead cells were stained with FVS (Fixable Viability Stains, BD Horizon™). The CD4^+^ cells were gated from the live cells and then the different subsets of T CD4^+^ cells were identified as: Treg (CD4^+^ CD25^+^ FoxP3^+^), Th17 (CD4^+^ IL-17a^+^), Th1 (CD4^+^ TNF-α^+^ or CD4^+^ IFN-γ^+^), and anti-inflammatory T cells (CD4^+^ IL-10^+^).


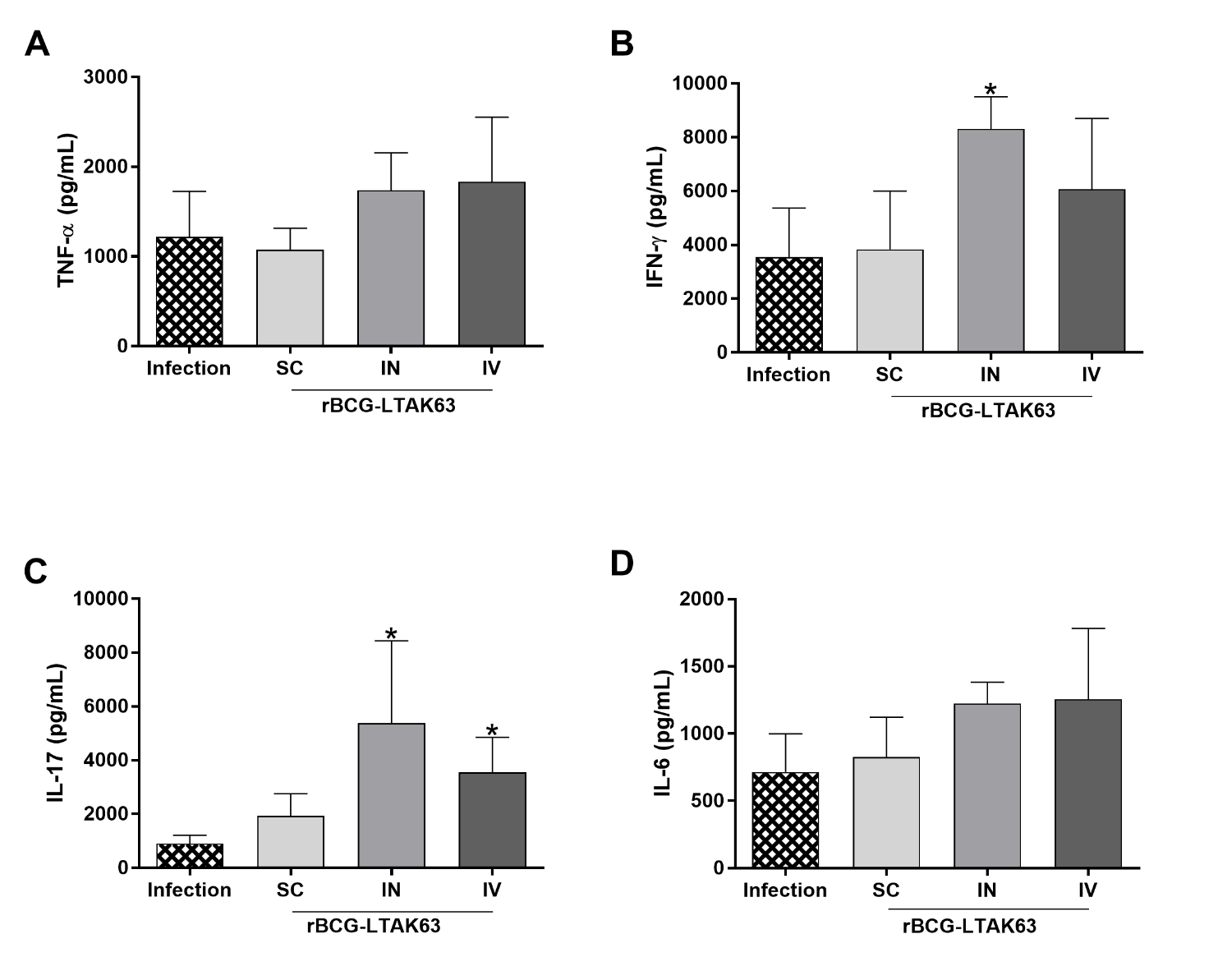


**Supplementary Figure 6.** Immunotherapy with two doses of rBCG-LTAK63 vaccine increases proinflammatory immune responses in *Mtb*-infected mice. BALB/c mice were infected with *Mtb* (500 CFU/IN); after 4 weeks the animals were treated with two doses of rBCG-LTAK63 by the SC, IN or IV routes, with four weeks intervals. Eight weeks after treatment, lungs were recovered, cells maintained in culture for 48 h (unstimulated) and the cytokine production analyzed in the supernatants with Cytometry Bead Array (A-D). Statistical differences were determined by one-way ANOVA with a Bonferroni test. * *p* values ≤ 0.05 were considered statistically significant. Asterisks over the columns refer to the comparison with the infection group. Results are represented by the means ± SD (n=5/group).
